# Ambient ultraviolet radiation as a cardioprotective factor: A global and regional analysis

**DOI:** 10.1002/hsr2.70065

**Published:** 2024-09-16

**Authors:** Wenpeng You

**Affiliations:** ^1^ Adelaide Medical School The University of Adelaide Adelaide South Australia Australia; ^2^ Heart and Lung, Royal Adelaide Hospital Adelaide South Australia Australia; ^3^ Adelaide Nursing School The University of Adelaide Adelaide South Australia Australia; ^4^ School of Nursing and Midwifery Western Sydney University Sydney New South Wales Australia

**Keywords:** ambient, cardiovascular disease (CVD), latitude, ultraviolet radiation, vitamin D

## Abstract

**Background:**

Ambient ultraviolet radiation (UVR) has been found to have a greater cardioprotective effect than previously believed. This study aimed to quantitatively measure the role of UVR in protecting against the progression of cardiovascular disease (CVD) in general on a global and regional scale.

**Methods:**

Population‐level data on UVR, CVD incidence, aging, economic affluence, CVD genetic background (indexed with the Biological State Index, I_bs_), obesity prevalence, and urbanization were collected and analysed. The correlation between UVR and CVD was examined using bivariate correlations, partial correlation, and stepwise multiple linear regression. Countries were grouped to investigate regional correlations between UVR and CVD, and Fisher's r‐to‐z transformation was used to compare correlation coefficients.

**Results:**

UVR showed a significant inverse correlation with CVD incidence rates in bivariate correlation analyses globally (*r* = − 0.775 and *r* = − 0.760, *p* < 0.001), as well as within high‐income (*r* = −0.704, *p* < 0.001) and low‐ and middle‐income countries (LMIC) (*r* = −0.851, *p* < 0.001). These correlations remained significant even after controlling for confounding variables (*r* = −0.689 to −0.812, *p* < 0.001). In stepwise regression models, UVR was found to be the most significant predictor of CVD incidence. The inverse correlation between UVR and CVD was stronger in LMICs compared to high‐income countries (*z* = −1.96, *p* < 0.050).

**Conclusions:**

Low ambient UVR may be a significant risk factor for the progression of CVD worldwide. The protective effect of UVR appears to be stronger in LMICs than in high‐income countries, suggesting a greater impact of UVR on CVD prevention in these regions. These findings emphasize the need for further research into the mechanisms underlying the cardioprotective effects of UVR and the development of public health strategies to mitigate CVD risk associated with low UVR exposure.

## 1 INTRODUCTION

Cardiovascular diseases (CVDs) are a wide range of disorders that affect the heart and blood vessels.^1^ They are the leading cause of global disease burden,^1^ with 55.5 million people diagnosed in 2019 alone, accounting for approximately one‐third of all deaths worldwide.^2^ This prevalence highlights the significant challenge that CVDs pose to global health, affecting both developed and developing nations.

Preventative measures targeting behavioral risk factors such as unhealthy diet, obesity, and physical inactivity are crucial for reducing the incidence of CVD.^3^ The risk factors for CVD are complex, involving a combination of cardiometabolic, behavioral, environmental, and social factors.^4^ Understanding the interplay between these factors is essential for understanding the complex causes of CVD.^5^, ^6^ Cardiometabolic factors like hypertension and dyslipidaemia directly impact cardiovascular health, while behavioral factors such as diet, physical activity, and tobacco use also play important roles.^4^ Environmental factors, including air quality and exposure to pollutants, further contribute to the risk, and social determinants such as socioeconomic status and healthcare access complicate the picture.^5^ A comprehensive understanding of these factors is necessary to design targeted strategies for preventing and managing CVD in diverse populations.

The relationship between sunlight exposure, specifically ultraviolet radiation (UVR), and cardiovascular health has been the subject of debate in observational studies.^7^, ^8^ Despite the known negative effects of UVR, historical records spanning over 6,000 years suggest that sunlight has been used therapeutically for cardiocirculatory and cardiovascular disorders.^9^ Natural heliotherapy is mentioned in the works of Hippocrates, ancient Roman health practices, and the practices of Islamic physicians in ancient Iran.^10^ Ambient sunlight plays a vital role in the production of vitamin D, which is essential for human health.^7^ When UVB light converts provitamin D3 into stable vitamin D3, it has a significant impact on our well‐being. The amount of UVR radiation we receive varies based on factors such as seasonal changes, skin pigmentation, and distance from the equator.^11^ Interestingly, there is a correlation between these variations in UVR radiation and changes in blood pressure.

Studies have shown that there is an inverse relationship between exposure to UVR radiation and both blood pressure and the incidence of CVD.^7^, ^12^ As we move further away from the equator, the levels of ambient UVR radiation decrease, coinciding with an increase in the risk of CVD. In ecological studies, researchers have observed seasonal variations in the prevalence of conditions like CVD, hypertension, and metabolic syndrome.^13^ Rates of these conditions tend to be lower in the summer but higher in the winter.^13^

This intricates connection between ambient UVR exposure, vitamin D levels, and the risk of CVD highlights the importance of understanding these factors so that we can develop effective preventive strategies.^14^, ^15^ Previous observational studies have shown an association between UVR radiation and CVD, but it's important to note that these studies may have been influenced by other factors. We need a more comprehensive analysis to truly understand this relationship further.^8^

**Figure 1:**
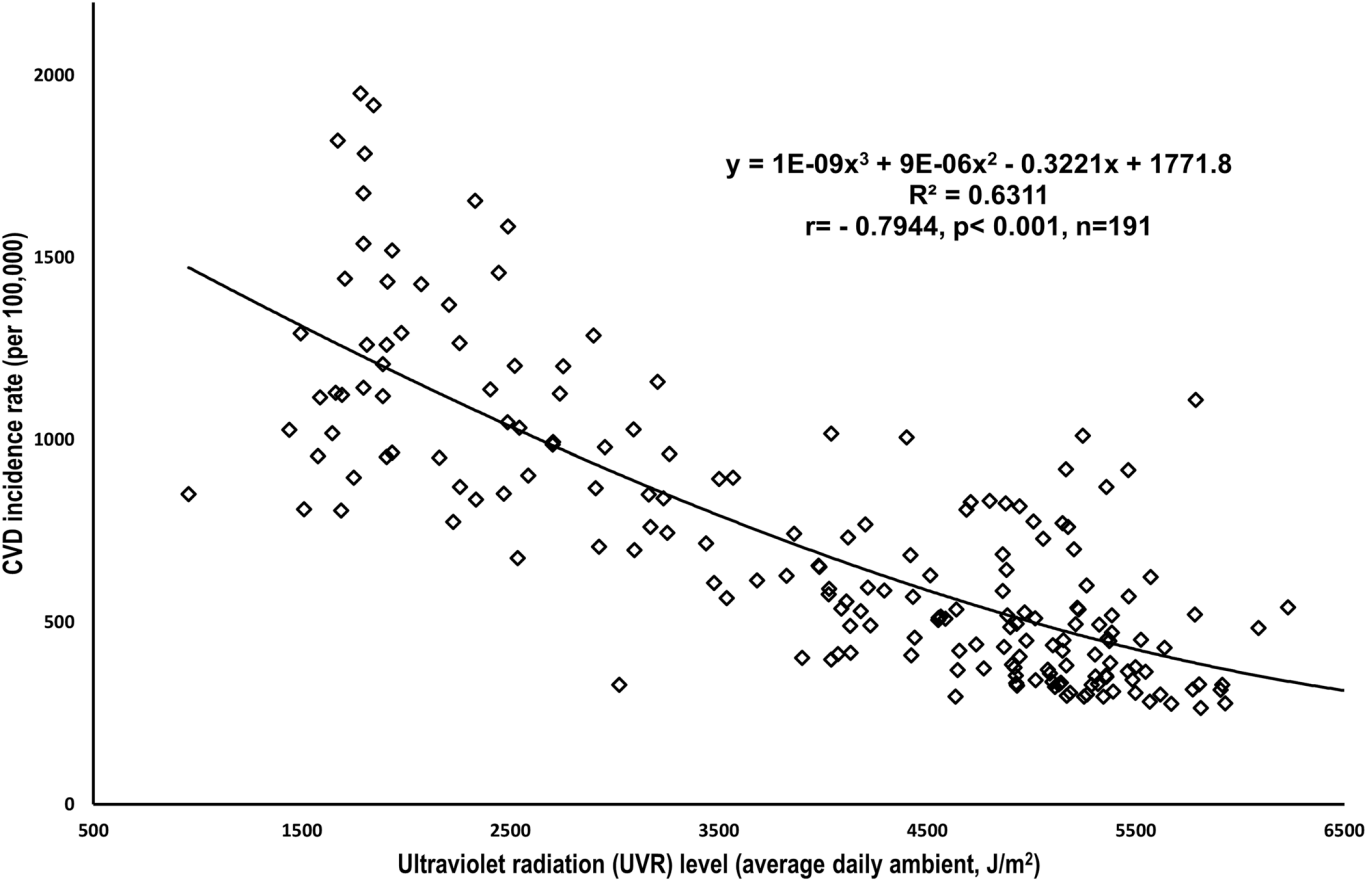
Plot to show the relationship between UV radiation and CVD incidence. Data source & definition: UV Radiation, expressed as the average daily ambient ultraviolet radiation level (in J/m^2^), the World Health Organization; CVD incidence, cardiovascular disease (CVD) incidence rate, the number of new cases per 100,000, the Institute for Health Metrics and Evaluation. Raw data were incorporated into the Excel for the generating the scatterplots.

Following previous observational studies with confounding residue, the UVR‐CVD relationship at population‐level can be depicted through the graph below^16^:

In the graph provided in Figure 1, we can see that UVR exposure statistically explains 63.11% of the variance in CVD, suggesting that it may play a main protective role against CVD‐related health issues. However, we need to approach these findings with caution,^16^ as we must consider other established factors like cardiometabolic, behavioral, environmental, and social risks that also contribute significantly to CVD.

Given the biases that have been observed in previous research, the aim of this study is to determine the independent influence of UVR exposure on the pathogenesis of CVD at a population level. To address potential biases in the UVR‐CVD relationship, this study first quantified the global predictive capacity of UVR exposure for CVD incidence. Then, it conducted an analysis to minimize the impact of confounding factors such as economic affluence, aging, genetic predisposition to CVD, obesity, and urbanization on the UVR‐CVD relationship. The ultimate result of this study is to reveal that UVR exposure is a significant and independent predictor for CVD incidence worldwide.

## 2 MATERIALS AND METHODS

### 2.1 Data collection and selection

This quantitative study gathered data from reputable international organizations and focused on country‐specific information. A list of WHO member countries was compiled to match the seven variables considered in the study. Throughout this article, the terms “population” and “country” are used interchangeably to refer to a geographic region or territory.^17^ This approach is adoped for ease of writing and to enhance readability.

1. The independent variable, the average daily ambient ultraviolet radiation (UVR) level, which encompasses solar radiation in the 280–400 nm range, was obtained as the independent variable from the WHO Global Health Observatory (GHO).^18^ The UVR level is quantified in joules per square meter (J/m²).^18^ In this study, population‐level exposure is represented by the annual ambient erythemal weighted UVR, which takes into account the biological effects on human skin by applying the Erythema Action Spectrum. This measure is expressed in joules effective per square meter (J/m²).^18^ It is calculated either from satellite data or by using a proxy such as latitudinal position.^18^
2. The dependent variable, the most recent CVD incidence rate (new cases per 100,000 population), was collected from the Institute for Health Metrics and Evaluation (IHME) at the University of Washington.^2^ In addition to exploring the predictive role of UVR exposure on total CVDs, this study also examined its influence on specific types of CVDs identified in previous research. The IHME provided incidence rates for all CVDs and 14 distinct types across various age groups. These types include ischemic heart disease, total stroke, ischemic stroke, intracerebral hemorrhage, subarachnoid hemorrhage, peripheral artery disease, atrial fibrillation and flutter, rheumatic heart disease, non‐rheumatic valvular heart disease (including non‐rheumatic degenerative mitral valve disease and non‐rheumatic calcific aortic valve disease), cardiomyopathy and myocarditis (including myocarditis), and endocarditis. All available data were integrated into the study for comprehensive analysis.
  In this study, we suggest that UVR generally helps protect against the development of CVDs. We have also chosen to explore the statistical predictive role of UVR because different types of CVDs have similar underlying mechanisms. While focusing on specific types of CVD could introduce bias, investigating the relationship between UVR and each individual CVD can still offer valuable evidence to support our main argument.
  Previous studies have suggested that the relationship between UVR and CVD may be influenced by other factors,^16^ so it is important to account for these potential confounders. This study included the following five variables that are recognized as major risk factors for CVD to isolate the independent effect of UVR exposure:
1. Aging, as indexed by life expectancy at age 65 years, was obtained from the United Nations.^19^ Age has been independently linked to the deterioration of cardiovascular health, significantly affecting the heart and arterial system.^20^, ^21^
2. Gross domestic product (GDP), converted to international dollars using purchasing power parity rates (GDP PPP), was extracted from the World Bank databank.^22^ Economic affluence impacts cardiovascular health through biological, behavioral, and psychosocial risk factors.^23^ It is also associated with education, employment, life expectancy, and access to healthcare, which all influence the accuracy of CVD diagnosis.^24^
3. The level of genetic background accumulation of CVD was measured using the Biological State Index (I_bs_), which ranges from 0 to 1.0. Higher I_bs_ values indicate reduced natural selection and have been associated with the accumulation of harmful genes/mutations [54].^25^ These genes/mutations are linked to conditions such as cancer, obesity, and type 1 diabetes. Including I_bs_ in the analysis helps address the impact of genetic predisposition on the UVR‐CVD correlation.
4. The prevalence of obesity, defined as the percentage of the population aged 18+ with a BMI ≥ 30 kg/m², was obtained from the WHO Global Health Observatory.^26^ Obesity increases the risk of CVD through factors such as atherosclerosis and comorbidities like hypertension, diabetes, and dyslipidaemia.^27^, ^28^, ^29^ It poses a multifactorial health challenge in terms of CVD prognosis.^27^
5. Urbanization, measured as the percentage of the population living in urban areas, was gathered from the World Bank.^22^ Urbanization has been linked to CVD morbidity and mortality, and recently, it has also been associated with vitamin D deficiency among CVD patients.^30^, ^31^ However, urban residents generally have better access to medical services, which aids in disease detection.

All of the above variables were extracted and matched with the WHO member list before being saved in Microsoft Excel® 2016 for further analysis. Each country was treated as an individual research subject, and the number of WHO member countries included for each variable may vary due to differences in data reporting.

### 2.2 Statistical analyses

An ecological study was conducted to examine the relationship between UVR exposure and the incidence of CVD at the population level.^32^, ^33^, ^34^, ^35^, ^36^, ^37^ To ensure accurate findings, several strategies were implemented: (1) different data analysis models were used for validation; (2) all seven variables were log‐transformed to reduce homoscedasticity; and (3) the correlation between family size and CVD incidence rate was assessed globally and regionally.

1. Bivariate Correlations: Pearson's and nonparametric analyses were performed to determine the strength and direction of the associations between UVR exposure and the incidence rates of total CVDs as well as 14 specific types.
2. Partial Correlation Analysis: Using the Pearson moment‐product approach, partial correlation was used to examine the independent relationships between UVR exposure and each of the 15 CVD incidence rates. This analysis considered confounding factors such as aging, GDP PPP, BMI, obesity, and urbanization to refine the examination of these relationships.
3. Independent Sample *t*‐test: This test was conducted to compare the means of each CVD variable between countries with higher and lower UVR levels. Countries were divided into two groups based on UVR exposure, using a cut point of 4420.5 J/m², which is the world average published by IHME. The hypothesis was that significantly different UVR exposure may lead to significantly different CVD incidence rates worldwide, as well as within high‐ and upper‐middle‐income countries (HUIC) and low‐ and middle‐income countries (LMIC).
4. Standard Multiple Linear Regression (stepwise): This analysis was used to identify variables that significantly predict CVD incidence rate. The independent variables included UVR exposure, aging, GDP PPP, BMI, obesity, and urbanization. A total of 15 CVD incidence variables were entered as dependent variables.
5. Pearson's *r* Correlation: To explore regional associations between UVR and CVD incidence, countries were categorized based on different criteria:

– World Bank Income Classifications: High‐income, upper‐middle‐income, low‐middle‐income, and low‐income. High‐income countries formed one grouping, while low‐ and middle‐income countries were combined into another (LMIC) as a new grouping. Pearson's r correlations were examined between UVR and CVD incidence in both groupings, and Fisher's r‐to‐z transformation was used to compare correlations.
– United Nations Classifications: Developed versus developing countries. Fisher's r‐to‐z transformation was used to compare correlation coefficients between UVR and CVD incidence in developed versus developing countries.
– Geographic and Socioeconomic Groupings: Seven groupings were analysed based on geographic distributions, per capita GDP levels, and cultural backgrounds: Asia Cooperation Dialog (ACD), Asia‐Pacific Economic Cooperation (APEC), the Arab World, countries with English as the official language, Latin America, Latin America and the Caribbean (LAC), and the Organization for Economic Co‐operation and Development (OECD).

SPSS v. 28 (SPSS Inc.) and Microsoft Excel 2016® were used for data analysis. The significance level was set at 0.05, with additional reporting at 0.01 and 0.001. The criteria for stepwise multiple linear regression analysis were a probability of F to enter ≤0.05 and a probability of F to remove ≥0.10.

## 3 RESULTS

Table 1 displays significant and consistent correlations between UVR and the incidence of CVD worldwide. These correlations are shown through both Pearson's r (*r* = −0.775, *p* < 0.001) and nonparametric analyses (*r* = −0.760, *p* < 0.001). The correlations hold true for both high‐income countries (HIC: *r* = −0.704, *p* < 0.001) and low‐ to middle‐income countries (LMIC: *r* = −0.851, *p* < 0.001). Even when controlling for five confounding variables in partial correlation analysis, the strong negative correlations persist (*r* = −0.689, −0.707, and −0.812, respectively, all *p* < 0.001), indicating that nations with higher UVR tend to have lower incidence rates of CVD, regardless of these factors.

**Table 1:** Bivariate (Pearson's *r*, nonparametric *ρ*) and partial correlation analyses to examine the relationships between ultraviolet radiation exposure and cardiovascular disease incidence rates (total and 13 types).

	All countries, *n* = 191						High‐ and Upper‐Income Countries (HUIC), n = 113						Low‐ and Middle‐Income Countries (LMIC), *n* = 78					
	Pearson's *r*		Nonparametric *ρ*		Partial correlation *r*		Pearson's *r*		Nonparametric *ρ*		Partial correlation r		Pearson's r		Nonparametric *ρ*		Partial correlation *r*	
	*r*	Sig. *p*	*ρ*	Sig. *p*	*r*	Sig. *p*	*r*	Sig. *p*	*ρ*	*p*	*r*	Sig. *p*	*r*	Sig. *p*	*ρ*	*n*	*r*	Sig. *p*
Cardiovascular diseases, total	−0.775	<0.001	−0.760	<0.001	−0.689	<0.001	−0.704	<0.001	−0.700	<0.001	−0.707	<0.001	−0.851	<0.001	−0.679	<0.001	−0.812	<0.001
Atrial fibrillation and flutter	−0.764	<0.001	−0.754	<0.001	−0.648	<0.001	−0.755	<0.001	−0.738	<0.001	−0.729	<0.001	−0.707	<0.001	−0.631	<0.001	−0.505	<0.001
Cardiomyopathy and myocarditis	−0.764	<0.001	−0.659	<0.001	−0.636	<0.001	−0.727	<0.001	−0.695	<0.001	−0.706	<0.001	−0.669	<0.001	−0.465	<0.001	−0.448	<0.001
Endocarditis	−0.502	<0.001	−0.473	<0.001	−0.083	0.298	−0.366	<0.001	−0.361	<0.001	−0.146	0.165	−0.291	<0.010	−0.144	0.209	0.055	0.668
Intracerebral hemorrhage	0.153	<0.050	0.143	<0.050	−0.155	<0.050	.206	<0.050	0.231	<0.050	−0.278	<0.010	−0.269	<0.050	−0.243	<0.050	−0.191	0.130
Ischemic heart disease	−0.647	<0.001	−0.657	<0.001	−0.517	<0.001	−0.507	<0.001	−0.469	<0.001	−0.479	<0.001	−0.785	<0.001	−0.670	<0.001	−0.769	<0.001
Ischemic stroke	−0.596	<0.001	−0.574	<0.001	−0.447	<0.001	−0.477	<0.001	−0.418	<0.001	−0.603	<0.001	−0.613	<0.001	−0.406	<0.001	−0.385	<0.010
Myocarditis	−0.764	<0.001	−0.659	<0.001	−0.636	<0.001	−0.727	<0.001	−0.695	<0.001	−0.706	<0.001	−0.669	<0.001	−0.465	<0.001	−0.448	<0.001
Nonrheumatic calcific aortic valve disease	−0.751	<0.001	−0.711	<0.001	−0.587	<0.001	−0.731	<0.001	−0.719	<0.001	−0.608	<0.001	−0.723	<0.001	−0.561	<0.001	−0.628	<0.001
Nonrheumatic degenerative mitral valve disease	−0.684	<0.001	−0.677	<0.001	−0.531	<0.001	−0.562	<0.001	−0.551	<0.001	−0.514	<0.001	−0.811	<0.001	−0.593	<0.001	−0.723	<0.001
Nonrheumatic valvular heart disease	−0.791	<0.001	−0.755	<0.001	−0.651	<0.001	−0.742	<0.001	−0.729	<0.001	−0.643	<0.001	−0.844	<0.001	−0.625	<0.001	−0.779	<0.001
Peripheral artery disease	−0.756	<0.001	−0.711	<0.001	−0.628	<0.001	−0.737	<0.001	−0.718	<0.001	−0.727	<0.001	−0.677	<0.001	−0.575	<0.001	−0.396	<0.001
Rheumatic heart disease	0.585	<0.001	0.601	<0.001	0.261	<0.001	0.443	<0.001	0.431	<0.001	0.166	0.115	0.603	<0.001	0.520	<0.001	0.374	<0.010
Stroke	−0.508	<0.001	−0.486	<0.001	−0.460	<0.001	−0.358	<0.001	−0.273	<0.010	−0.607	<0.001	−0.636	<0.001	−0.484	<0.001	−0.428	<0.001
Subarachnoid hemorrhage	−0.633	<0.001	−0.656	<0.001	−0.441	<0.001	−0.526	<0.001	−0.579	<0.001	−0.507	<0.001	−0.654	<0.001	−0.536	<0.001	−0.490	<0.001
Ageing e(65)	−0.560	<0.001	−0.550	<0.001	‐	‐	−0.443	<0.001	−0.455	<0.001	‐	‐	−0.375	<0.001	−0.315	<0.010	‐	‐
GDP PPP	−0.612	<0.001	−0.629	<0.001	‐	‐	−0.532	<0.001	−0.541	<0.001	‐	‐	−0.574	<0.001	−0.439	<0.001	‐	‐
I_bs_	−0.467	<0.001	−0.630	<0.001	‐	‐	−0.329	<0.001	−0.568	<0.001	‐	‐	−0.489	<0.001	−0.497	<0.001	‐	‐
Obesity %	−0.294	<0.001	−0.329	<0.001	‐	‐	−0.008	0.939	0.029	0.767	‐	‐	−0.231	<0.050	−0.239	<0.050	‐	‐
Urbanization	−0.439	<0.001	−0.466	<0.001	‐	‐	−0.323	<0.001	−0.292	<0.010	‐	‐	−0.253	<0.050	−0.163	0.156	‐	‐

*Note*: Significance levels: *p < 0.05, ***p < 0.01, ***p < 0.001. Control variable: Data source & definition: Ultraviolet Radiation (UVR), expressed as the average daily ambient ultraviolet radiation level (in J/m^2^), the World Health Organization; Cardiovascular disease (CVD) incidence rate, the number of newly diagnosed cases per 100,000 in 2019, the Institute for Health Metrics and Evaluation; Ageing indexed with life expectancy at 65 year old in 2014, United Nations; Per capita GDP PPP, measured with the per capita purchasing power parity (PPP) value of all final goods and services produced within a territory in 2019, the World Bank; I_bs_ indexing the detrimental CVD genetic background accumulation in the past decades, downloaded from previous publications.^33^ Obesity prevalence, measured with the percentage of population aged 18+ with BMI equal to or over 30 kg/m^2^ in 2014, the World Health Organization. Urbanization, measured with the percentage of population living in urban area in 2019, the World Bank. All the data were log‐transformed for correlation analysis in SPSS v 28.

Regarding specific types of CVD, Table 1 demonstrates moderate to strong and statistically significant inverse relationships between UVR and each of the 13 types examined, except for intracerebral hemorrhage and rheumatic heart disease.

Overall, this bivariate correlation analysis highlights moderate to strong inverse correlations between UVR and incidence rates of CVD. These correlations remain statistically significant even after considering potential confounders such as aging, GDP per capita (PPP), obesity, smoking prevalence (I_bs_), and urbanization (Table 1). This confirms the inclusion of these variables as controls when assessing the association between UVR and CVD incidence rates.

Table 2 shows that when using a cut‐off of 4420.5 J/m² (the global median for ultraviolet radiation), independent sample t‐tests revealed significant differences in mean values between country groups exposed to higher versus lower levels of ultraviolet radiation worldwide (*t* = 21.074, *p* < 0.001). This was true for both high‐ and low‐ and middle‐income ountries (HUIC: *t* = 15.140, *p* < 0.001; LMIC: *t* = 13.136, *p* < 0.001). Interestingly, countries with higher ultraviolet radiation levels above the cut‐off point had significantly lower overall incidence rates of CVD compared to those with lower ultraviolet radiation worldwide (*t* = −11.921, *p* < 0.010). This trend was observed within both HUIC (*t* = −7.373, *p* < 0.010) and LMIC (*t* = −7.618, *p* < 0.010). However, this disparity was not observed in cases of intracerebral hemorrhage and rheumatic heart disease. The findings presented in Table 2 suggest that variations in ultraviolet radiation exposure may significantly influence the incidence rates of different types of cardiovascular disease.

**Table 2:** Independent samples *t*‐test to compare the differences between the means in two country groups with the cut point of 4420.5 J/m^2^(median of ultraviolet radiations).

	All countries, *n* = 191, cut point 4420.5				High‐ and upper‐income Countries (HUIC), *n* = 113, cut point 4420.5				Low‐ and middle‐income Countries (LMIC), *n* = 68, cut point 4420.5			
	UVR				UVR				UVR			
	Mean >4420.5	Mean <4420.5	Mean difference, *t*	Sig. *p*	Mean > 4420.5	Mean <4420.5	Mean difference *t*	Sig. *p*	Mean >4420.5	Mean (<4420.5)	Mean difference *t*	Sig. *p*
UVR	5220.52	2791.07	21.074	<0.01	5185.66	2609.77	15.140	<0.01	5247.44	3386.76	13.136	<0.010

	CVD incidence rate				CVD incidence rate				CVD incidence rate			
	Mean > 4420.5,	Mean <4420.5	Mean difference *t*	Sig. *p*	Mean > 4420.5	Mean <4420.5	Mean difference *t*	Sig. *p*	Mean >4420.5	Mean (<4420.5)	Mean difference *t*	Sig. *p*
Cardiovascular diseases, total	479.51	973.68	−11.921	<0.001	603.72	1031.81	−7.373	<0.001	383.63	782.68	−7.618	<0.001
Atrial fibrillation and flutter	26.82	82.02	−11.483	<0.001	37.38	91.85	−7.973	<0.001	18.66	49.73	−5.984	<0.001
Cardiomyopathy and myocarditis	12.42	18.60	−10.006	<0.001	14.04	20.19	−7.329	<0.001	11.17	13.38	−3.866	<0.001
Endocarditis	12.28	19.22	−5.448	<0.001	17.47	22.03	−2.572	<0.050	8.28	10.00	−2.082	<0.050
Intracerebral hemorrhage	43.23	38.08	1.497	0.136	44.04	34.09	2.274	<0.050	42.60	51.17	−1.385	0.170
Ischemic heart disease	172.70	424.43	−9.736	<0.001	249.20	433.21	−4.894	<0.001	113.64	395.56	−8.389	<0.001
Ischemic stroke	62.89	113.28	−7.365	<0.001	78.88	120.07	−3.985	<0.001	50.55	90.97	−5.257	<0.001
Myocarditis	12.42	18.60	−10.006	<0.001	14.04	20.19	−7.329	<0.001	11.17	13.38	−3.866	<0.001
Nonrheumatic calcific aortic valve disease	1.63	20.42	−7.923	<0.001	3.21	24.35	−6.111	<0.001	0.40	7.50	−2.406	<0.050
Nonrheumatic degenerative mitral valve disease	1.88	19.88	−6.417	<0.001	3.03	22.82	−4.274	<0.001	0.99	10.21	−4.895	<0.001
Nonrheumatic valvular heart disease	3.51	40.29	−8.174	<0.001	6.24	47.17	−5.923	<0.001	1.39	17.70	−3.506	<0.001
Peripheral artery disease	78.44	198.14	−10.738	<0.001	106.78	226.76	−7.903	<0.001	56.57	104.10	−2.105	<0.050
Rheumatic heart disease	57.03	19.28	9.946	<0.001	35.77	14.17	5.834	<0.001	73.43	36.05	−4.582	<0.001
Stroke	116.32	171.70	−5.982	<0.001	136.83	176.42	−2.875	<0.010	100.48	156.17	5.968	<0.001
Subarachnoid hemorrhage	10.20	20.34	−6.831	<0.001	13.92	22.26	−3.639	<0.001	7.33	14.03	−4.667	<0.001

*Note*: Significance levels: *p < 0.05, ***p < 0.01, ***p < 0.001. Data source and definition: Ultraviolet radiation (UVR), expressed as the average daily ambient ultraviolet radiation level (in J/m^2^), the World Health Organization; Cardiovascular disease (CVD) incidence rate, the number of newly diagnosed cases per 100,000 in 2019, the Institute for Health Metrics and Evaluation. All the data were not log‐transformed for mean compassion in SPSS v 28.

Table 3 presents the results of the stepwise linear regression model, which highlights UVR as the most significant predictor of total CVD incidence globally. This finding holds true for both high‐income and low‐ and middle‐income countries, with adjusted R^2^ values of 0.629, 0.519, and 0.748 respectively. The analysis shows a strong inverse relationship between UVR and CVD incidence rates worldwide, as well as in both high‐income and low‐ and middle‐income countries. The beta coefficients for these relationships are −0.607, −0.716, and −0.709 respectively, with p‐values less than 0.010.

**Table 3:** Stepwise multiple linear regression to identify the significant predictors of cardiovascular disease incidence rates (cardiovascular disease incidence rates [total and 14 types]).

	All Countries, n = 191				High‐ and Upper‐middle Income Countries (HUIC), n = 113				Low‐ and Low‐middle Income Countries (LMIC), n = 68			
	Independent predictor	Adjusted R^2^	Beta	Sig. *p*	Independent predictor	Adjusted R^2^	Beta	Sig. *p*	Independent predictor	Adjusted R^2^	Beta	Sig. *p*
Cardiovascular diseases, total	UV radiation	0.629	−0.607	<0.001	UV radiation	0.519	−0.716	<0.001	UV radiation	0.748	−0.709	<0.001
	I_bs_	0.742	0.387	<0.001	Urbanization	0.561	−0.233	<0.001	I_bs_	0.829	0.327	<0.001
	Ageing e(65)	Insignificant			I_bs_	0.605	0.231	<0.001	Ageing e(65)	Insignificant		
	GDP PPP	Insignificant			Ageing e(65)	Insignificant			GDP PPP	Insignificant		
	Obesity %	Insignificant			GDP PPP	Insignificant			Obesity %	Insignificant		
	Urbanization	Insignificant			Obesity %	Insignificant			Urbanization	Insignificant		
Atrial fibrillation and flutter	UV radiation	0.597	−0.559	<0.001	UV radiation	0.577	−0.734	<0.001	GDP PPP	0.485	0.243	<0.050
	I_bs_	0.747	0.443	<0.001	I_bs_	0.628	0.255	<0.001	UV radiation	0.605	−0.384	<0.001
	Ageing e(65)	Insignificant			Urbanization	0.658	−0.193	<0.010	I_bs_	0.664	0.347	<0.001
	GDP PPP	Insignificant			Ageing e(65)	Insignificant			Ageing e(65)	Insignificant		
	Obesity %	Insignificant			GDP PPP	Insignificant			Obesity %	Insignificant		
	Urbanization	Insignificant			Obesity %	Insignificant			Urbanization	Insignificant		
Cardiomyopathy and myocarditis	UV radiation	0.589	−0.505	<0.001	UV radiation	0.523	−0.617	<0.001	GDP PPP	0.442	0.293	<0.050
	Ageing e(65)	0.759	0.444	<0.001	Ageing e(65)	0.658	0.401	<0.001	UV radiation	0.535	−0.369	<0.001
	Obesity %	0.766	−0.138	<0.010	Obesity %	0.693	−0.177	<0.010	I_bs_	0.573	0.335	<0.010
	I_bs_	0.772	0.138	<0.050	Urbanization	0.706	−0.136	<0.050	Obesity %	0.607	−0.210	<0.050
	GDP PPP	Insignificant			GDP PPP	Insignificant			Ageing e(65)	Insignificant		
	Urbanization	Insignificant			I_bs_	Insignificant			Urbanization	Insignificant		
Endocarditis	Ageing e(65)	0.616	0.697	<0.001	Ageing e(65)	0.440	5.325	<0.001	GDP PPP	0.216	0.374	<0.010
	Obesity %	0.657	0.224	<0.001	I_bs_	0.485	3.054	0.003	Urbanization	0.253	0.241	<0.050
	UV radiation	Insignificant			UV radiation	Insignificant			UV radiation	Insignificant		
	GDP PPP	Insignificant			GDP PPP	Insignificant			Ageing e(65)	Insignificant		
	I_bs_	Insignificant			Obesity %	Insignificant			I_bs_	Insignificant		
	Urbanization	Insignificant			Urbanization	Insignificant			Obesity %	Insignificant		
Intracerebral hemorrhage	Ageing e(65)	0.203	−0.818	<0.001	Ageing e(65)	0.307	−0.630	<0.001	UV radiation	Insignificant		
	I_bs_	0.312	0.601	<0.001	Urbanization	0.384	−0.205	0.019	Ageing e(65)	Insignificant		
	Obesity %	0.338	−0.203	<0.010	I_bs_	0.416	0.305	0.002	GDP PPP	Insignificant		
	UV radiation	Insignificant			Obesity %	0.459	−0.262	0.001	I_bs_	Insignificant		
	GDP PPP	Insignificant			UV radiation	0.478	−0.238	0.007	Obesity %	Insignificant		
	Urbanization	Insignificant			GDP PPP	0.500	−0.263	<0.050	Urbanization	Insignificant		
Ischemic heart disease	UV radiation	0.460	−0.472	<0.001	UV radiation	0.280	−0.501	<0.001	UV radiation	0.714	−0.724	<0.001
	I_bs_	0.581	0.327	<0.001	I_bs_	0.322	0.227	<0.010	I_bs_	0.761	0.255	<0.001
	Obesity %	0.595	0.151	<0.050	Obesity %	0.349	0.202	<0.050	Ageing e(65)	Insignificant		
	Ageing e(65)	Insignificant			Urbanization	0.374	−0.185	<0.050	GDP PPP	Insignificant		
	GDP PPP	Insignificant			Ageing e(65)	Insignificant			Obesity %	Insignificant		
	Urbanization	Insignificant			GDP PPP	Insignificant			Urbanization	Insignificant		
Ischemic stroke	UV radiation	0.367	−0.483	<0.001	UV radiation	0.235	−0.604	<0.001	UV radiation	0.354	−0.438	<0.001
	I_bs_	0.468	0.495	<0.001	Ageing e(65)	0.302	−0.575	<0.001	I_bs_	0.437	0.342	<0.010
	Ageing e(65)	0.483	−0.207	<0.050	I_bs_	0.367	0.402	<0.001	Ageing e(65)	Insignificant		
	GDP PPP	Insignificant			Obesity %	0.421	−0.251	0.003	GDP PPP	Insignificant		
	Obesity %	Insignificant			GDP PPP	Insignificant			Obesity %	Insignificant		
	Urbanization	Insignificant			Urbanization	Insignificant			Urbanization	Insignificant		
Myocarditis	UV radiation	0.589	−0.505	<0.001	UV radiation	0.523	−0.617	<0.001	GDP PPP	0.442	0.293	<0.050
	Ageing e(65)	0.759	0.444	<0.001	Ageing e(65)	0.658	0.401	<0.001	UV radiation	0.535	−0.369	<0.001
	Obesity %	0.766	−0.138	<0.010	Obesity %	0.693	−0.177	<0.010	I_bs_	0.573	0.335	<0.010
	I_bs_	0.772	0.138	<0.050	Urbanization	0.706	−0.136	<0.050	Obesity %	0.607	−0.210	<0.050
	GDP PPP	Insignificant			GDP PPP	Insignificant			Ageing e(65)	Insignificant		
	Urbanization	Insignificant			I_bs_	Insignificant			Urbanization	Insignificant		
Nonrheumatic calcific aortic valve disease	GDP PPP	0.676	0.343	<0.001	UV radiation	0.526	−8.573	<0.001	UV radiation	0.574	−0.572	<0.001
	UV radiation	0.783	−0.405	<0.001	Ageing e(65)	0.608	4.548	<0.001	GDP PPP	0.626	0.251	<0.010
	Ageing e(65)	0.793	0.209	<0.010	GDP PPP	Insignificant			Obesity %	0.647	0.170	<0.050
	Obesity %	0.800	0.106	<0.050	I_bs_	Insignificant			Ageing e(65)	Insignificant		
	I_bs_	Insignificant			Obesity %	Insignificant			I_bs_	Insignificant		
	Urbanization	Insignificant			Urbanization	Insignificant			Urbanization	Insignificant		
Nonrheumatic degenerative mitral valve disease	UV radiation	0.473	−0.528	<0.001	UV radiation	0.307	−0.575	<0.001	UV radiation	0.670	−0.686	<0.001
	I_bs_	0.557	0.336	<0.001	Obesity %	0.336	−0.263	<0.010	I_bs_	0.726	0.280	<0.001
	Ageing e(65)	Insignificant			Ibs	0.366	0.369	<0.001	Ageing e(65)	Insignificant		
	GDP PPP	Insignificant			Ageing e(65)	0.412	−0.305	<0.010	GDP PPP	Insignificant		
	Obesity %	Insignificant			GDP PPP				Obesity %	Insignificant		
	Urbanization	Insignificant			Urbanization				Urbanization	Insignificant		
Nonrheumatic valvular heart disease	UV radiation	0.626	−0.502	<0.001	UV radiation	0.528	−0.652	<0.001	UV radiation	0.729	−0.717	<0.001
	GDP PPP	0.762	0.386	<0.001	I_bs_	0.578	0.258	<0.001	I_bs_	0.790	0.287	<0.001
	I_bs_	0.778	0.201	<0.001	Obesity %	0.598	−0.155	<0.050	Ageing e(65)	Insignificant		
	Urbanization	0.782	−0.102	<0.050	Ageing e(65)				GDP PPP	Insignificant		
	Ageing e(65)	Insignificant			GDP PPP				Obesity %	Insignificant		
	Obesity %	Insignificant			Urbanization				Urbanization	Insignificant		
Peripheral artery disease	UV radiation	0.584	−0.463	<0.001	UV radiation	0.555	−0.661	<0.001	I_bs_	0.546	0.422	<0.001
	Ageing e(65)	0.755	0.304	<0.001	Ageing e(65)	0.638	0.364	<0.001	UV radiation	0.645	−0.280	<0.010
	I_bs_	0.789	0.282	<0.001	Urbanization	0.669	−0.198	<0.010	GDP PPP	0.675	0.272	<0.010
	GDP PPP	Insignificant			GDP PPP	Insignificant			Ageing e(65)	Insignificant		
	Obesity %	Insignificant			I_bs_	Insignificant			Obesity %	Insignificant		
	Urbanization	Insignificant			Obesity %	Insignificant			Urbanization	Insignificant		
Rheumatic heart disease	GDP PPP	0.576	−0.466	<0.001	GDP PPP	0.363	−0.325	<0.001	GDP PPP	0.443	−0.280	<0.050
	UV radiation	0.603	0.209	<0.001	I_bs_	0.410	−0.299	<0.001	Ageing e(65)	0.501	−0.331	<0.010
	Ageing e(65)	0.612	−0.196	<0.050	Urbanization	0.467	−0.286	<0.001	UV radiation	0.560	0.310	<0.010
	I_bs_	Insignificant			UV radiation	Insignificant			I_bs_	Insignificant		
	Obesity %	Insignificant			Ageing e(65)	Insignificant			Obesity %	Insignificant		
	Urbanization	Insignificant			Obesity %	Insignificant			Urbanization	Insignificant		
Stroke	UV radiation	0.276	−6.860	<0.001	UV radiation	0.153	−0.628	<0.001	UV radiation	0.361	−0.419	<0.001
	I_bs_	0.359	7.092	<0.001	Ageing e(65)	0.268	−0.562	<0.001	I_bs_	0.472	0.392	<0.001
	Ageing e(65)	0.442	−5.021	<0.001	I_bs_	0.353	0.477	<0.001	Ageing e(65)	Insignificant		
	GDP PPP				Obesity %	0.451	−0.340	<0.001	GDP PPP	Insignificant		
	Obesity %				GDP PPP	0.476	−0.264	<0.050	Obesity %	Insignificant		
	Urbanization				Urbanization				Urbanization	Insignificant		
Subarachnoid hemorrhage	I_bs_	0.624	0.626	<0.001	I_bs_	0.363	0.502	<0.001	I_bs_	0.615	0.613	<0.001
	UV radiation	0.713	−0.342	<0.001	UV radiation	0.479	−0.380	<0.001	UV radiation	0.711	−0.360	<0.001
	Ageing e(65)	Insignificant			Obesity %	0.558	−0.288	<0.001	Ageing e(65)	Insignificant		
	GDP PPP	Insignificant			Ageing e(65)	Insignificant			GDP PPP	Insignificant		
	Obesity %	Insignificant			GDP PPP	Insignificant			Obesity %	Insignificant		
	Urbanization	Insignificant			Urbanization	Insignificant			Urbanization	Insignificant		

*Note*: Significance levels: * *p* < 0.05, *** *p* < 0.01, *** *p* < 0.001. Data source and definition: Ultraviolet radiation (UVR), expressed as the average daily ambient ultraviolet radiation level (in J/m^2^), the World Health Organization; Cardiovascular disease (CVD) incidence rate, the number of newly diagnosed cases per 100,000 in 2019, the Institute for Health Metrics and Evaluation; Ageing indexed with life expectancy at 65 year old in 2014, United Nations; Per capita GDP PPP, measured with the per capita purchasing power parity (PPP) value of all final goods and services produced within a territory in 2019, the World Bank; I_bs_ indexing the detrimental CVD genetic background accumulation in the past decades, downloaded from previous publications^34^; Obesity prevalence, measured with the percentage of population aged 18+ with BMI equal to or over 30 kg/m^2^ in 2014, the World Health Organization. Urbanization, measured with the percentage of population living in urban area in 2019, the World Bank. All the data were log‐transformed for correlation analysis in SPSS v 28.

With the exception of endocarditis and intracerebral hemorrhage, UVR is found to be a significant predictor for all 12 types of CVD analysed. However, it is not always the most influential predictor. Additionally, there is a significant correlation between UVR and each of these 12 types of CVD.

Table 4 presents the correlation between UVR and CVD incidence across different country clusters, using Pearson's *r*. Generally, there was a consistent negative correlation between UVR and CVD incidence within each country grouping. However, the strength and significance of these correlations may vary depending on sample size and the predictive role of UVR in CVD incidence.

**Table 4:** Ultraviolet radiation (UVR) level determining total cardiovascular disease incidence rate in different country groupings.

Country groupings	Pearson *r*	*p*
World Bank income classifications		
Low Income, *n* = 30	−0.684**	<0.001
Low Middle Income, *n* = 49	−0.846**	<0.001
Upper Middle Income, *n* = 51	−0.740**	<0.001
Low‐ and middle‐income countries (LMIC), *n* = 130	−0.785**	<0.001
High Income, *n* = 60	−0.633**	<0.001
Fisher r‐to‐z transformation	LMIC versus High: *z* = − 1.96, *p* < 0.050	
United Nations common practice		
Developed, *n* = 44	0.025	0.872
Developing, *n* = 146	−0.582**	<0.001
Fisher r‐to‐z transformation	LMIC versus High: z = − 3.90, *p* < 0.001	
Countries grouped with various factors		
Asia Cooperation Dialog, *n* = 33	−0.776**	<0.001
Asia‐Pacific Economic Cooperation, *n* = 19	−0.900**	<0.001
Arab World, *n* = 21	−0.872**	<0.001
English as Official Language, *n* = 54	−0.441**	<0.001
Latin America, *n* = 20	−0.632**	<0.010
Latin America and Caribbean, *n* = 33	−0.124	0.494
Organization for Economic Co‐operation and Development, *n* = 38	−0.598**	<0.001

*Note*: Pearson r and nonparametric correlations within country groupings were reported. Data source & definition: Ultraviolet Radiation (UVR), expressed as the average daily ambient ultraviolet radiation level (in J/m^2^), the World Health Organization; Cardiovascular disease (CVD) incidence rate, the number of new cases per 100,000, the Institute for Health Metrics and Evaluation. All the data were log‐transformed for correlation analysis.

By applying Fisher's r‐to‐z transformation, it was found that the correlation between UVR and CVD incidence was significantly stronger in low‐ and middle‐income countries (LMIC) compared to high‐income countries (z = −1.96, *p* < 0.050). Additionally, the correlation in developing countries was significantly stronger than in developed countries (z = −3.90, *p* < 0.001). These results suggest that UVR may have a more pronounced impact on CVD incidence in LMIC than in high‐income countries (Table 4).

## 4 DISCUSSION

CVDs are a significant public health challenge influenced by various factors, including exposure to UVR. This study examines the relationship between UVR exposure and the incidence of 14 specific types of CVD [53], highlighting UVR as a notable risk factor in their development.^38^

1. UVR exposure is significantly correlated with a reduction in the overall incidence of CVD. When accounting for confounding factors such as aging, economic status, obesity, and urbanization, UVR explains 63.11% of the variance. This statistical association underscores the protective role of UVR across different types of CVD.
2. However, after adjusting for established confounders like aging and economic status, the impact of UVR on the incidence of total CVD slightly diminishes. Stepwise linear regression reveals that UVR explains 36.84% of the variance, while partial correlation analysis attributes 47.47% to UVR, indicating a persistent albeit reduced association.
3. Interestingly, the protective effect of UVR appears to be more pronounced in developing countries compared to developed ones, suggesting varying impacts across different geographic and economic contexts.

Throughout human evolution, there has been significant exposure to sunlight, approximately half of each day.^39^ While research has highlighted the adverse effects of UVR, such as skin cancer and photo‐aging, recent insights challenge conventional wisdom.^40^ Contrary to prevailing beliefs, reduced UVR exposure, rather than increased exposure, may contribute to the development of skin cancer, prompting a re‐evaluation of the complex relationship between UVR and human health.^36^, ^41^

Ultraviolet radiation from sunlight is believed to provide cardiovascular benefits and has been used for over 6,000 years in the treatment of cardiocirculatory disorders.^42^, ^43^ However, there is no scientific consensus on the consistent cardioprotective effects of UVR.

One established mechanism links UVR exposure to the synthesis of vitamin D in the skin,^44^ which helps protect against CVD and high blood pressure. Another suggests that UVR stimulates the conversion of skin nitrogen oxides to nitric oxide (NO), promoting coronary vasodilation and exhibiting cardioprotective and antihypertensive properties.^8^ Although UV‐A radiation does not promote vitamin D synthesis,^45^ it has been associated with lower blood pressure.^40^ Moreover, while greater UVR exposure may degrade bioactive folate metabolites,^46^ including 5‐methyltetrahydrofolate, known for its cardioprotective properties, this study focuses on the role of natural ambient UVR in maintaining and improving cardiovascular health, without delving into the specifics of the UVR spectrum and its controversial impact on vitamin D and folate synthesis.

Scragg conducted an ecological study that found a possible link between seasonal variations in UVR and the observed patterns of CVD.^47^ In contrast to this descriptive observational approach, this study took a quantitative approach using population‐level data. As the lead author of a recent systematic review, Scragg et al. raised concerns about previous studies that linked UVR to CVD, suggesting that other influencing risk factors may have confounded the results.^47^ This study addresses this concern by using partial correlation and stepwise linear regression to independently investigate the role of UVR in predicting CVD incidence globally.

Many studies have examined the statistical relationship between UVR and blood pressure.^12^ However, it is important to note that while high blood pressure is a significant risk factor for CVD,^48^, ^49^ it is not a disease in itself. Blood pressure readings can be influenced by various factors, such as medical interventions, emotional states, physical activities, and environmental conditions. Previous research has mainly focused on high blood pressure as a risk factor and has often overlooked the fact that low blood pressure can also pose risks for CVD.^48^, ^49^ Therefore, using blood pressure as a dependent variable to explore the role of UVR exposure in predicting CVD may not provide accurate insights.

The presence of biases in previous research may have led to null or positive correlations between UVR and CVD. For example, in a study involving 17,773 American stroke patients over the age of 45,^50^ Kent et al. did not observe a correlation between daily ambient solar radiation and brachial blood pressure.^50^ Similarly, Scragg et al. conducted a clinical trial with 119 individuals in New Zealand who had low vitamin D levels and found that ultraviolet exposure did not lead to a reduction in blood pressure.^51^ These studies may have been affected by biases such as limited latitude range exposure and biased blood pressure readings from subjects who were already unhealthy.^51^ Similar biases were also observed in a study involving male cyclists and triathletes in the United Kingdom.^52^

A quartile correlation analysis showed a weak positive correlation between UVR and CVD mortality in a cohort study of individuals aged 51‐70 in the United States.^53^ However, this method only measured relationships between quartiles and not individual data points, which limits its accuracy. Additionally, using CVD mortality as a measure of UVR exposure's role as a risk factor may be flawed, as CVD is preventable and involves distinct phases before and after diagnosis.^54^ This improper measure may bias the findings suggesting a detrimental role of UVR in the development of CVD. The inverse correlation between UVR and CVD incidence has been observed in various countries, which has led to an examination of UVR's effects on CVD in LMICs and high‐income countries (1). This study shows that the correlations between UVR and CVD incidence are much stronger in LMICs compared to high‐income countries. This difference may be influenced by economic factors and disparities in healthcare education between these regions.

### 4.1 Strength and limitation of this study

Population‐level data, including second‐hand data, are utilized in this study despite potential random errors during collection and aggregation. This enhances the repeatability of our analysis by minimizing subjective biases often found in individual‐based quantitative studies. However, several limitations should be acknowledged:

First, this study is quantitative and focuses on correlating variables, which does not imply causation even if associations are observed concurrently.

Second, as a population‐level study, each population serves as the research unit, potentially leading to the intrinsic ecological fallacy in data analysis. Therefore, correlations identified at the country level may not necessarily be applicable at the individual level.

Third, the incidence of CVD is influenced by the availability of formal medical diagnoses, closely tied to economic prosperity and urbanization levels. The country‐specific CVD incidence rates from the University of Washington data set may be incomplete, particularly in LMICs. Economic affluence (indexed by GDP PPP) and urban advantages (measured by urbanization rates) have been integrated into our analyses to mitigate potential confounding effects on CVD incidence accuracy, yet residual confounders may persist.

Fourth, the total population‐level exposure to UVR is included as an independent variable to assess its statistical role in determining CVD incidence rates. However, real‐life UVR exposure is influenced by various behavioral factors, such as sunglasses usage, sunblock application, outdoor recreational activities, clothing choices, and duration of outdoor exposure. These factors significantly modify UVR exposure levels, but their exact impact has not been quantified, posing a challenge in incorporating these potential confounders into the statistical models in this study. To address this limitation, future studies could consider innovative methods, such as using Satellite Earth Observation for near real‐time monitoring of UV‐A solar radiation,^55^ which offers more precise measurements.^55^, ^56^ These advancements could support further research into the relationship between personal solar exposure and its implications for cardiovascular health.

## 5 CONCLUSION

Ambient UVR may play a significant and independent role in protecting against CVD progression worldwide. This protective effect appears to be more prominent in LMIC. However, since there is a lack of evidence to support the cardioprotective role of vitamin D supplementation, it is worth exploring whether ambient UVR can also protect against CVD independently of vitamin D supplementation.

## AUTHOR CONTRIBUTIONS

**Wenpeng You**: Conceptualization; Investigation; Funding acquisition; Writing—original draft; Methodology; Validation; Visualization; Writing—review and editing; Software; Formal analysis; Project administration; Data curation; Resources.

## CONFLICT OF INTEREST STATEMENT

The sole author declares that there is no conflict of interest regarding the publication of this article. The author has read and approved the final version of the manuscript (corresponding author: WY) had full access to all of the data in this study and takes complete responsibility for the integrity of the data and the accuracy of the data analysis.

## ETHICS STATEMENT

All variables used in this study were collected from reputable sources, such as international organizations like United Nations agencies and the Institute for Health Metrics and Evaluation (IHME). The data set comprises solely of non‐identifiable, pre‐existing data about human subjects. It is not possible to trace individual persons or their communities using this data. Therefore, the study poses minimal ethical risk and does not necessitate ethical approval or written informed consent from participants.

## TRANSPARENCY STATEMENT

The lead author Wenpeng You affirms that this manuscript is an honest, accurate, and transparent account of the study being reported; that no important aspects of the study have been omitted; and that any discrepancies from the study as planned (and, if relevant, registered) have been explained.

## Data Availability

The data used for this study were obtained from publicly available repositories hosted by United Nations (UN) Agencies and the Institute for Health Metrics and Evaluation at the University of Washington. Our use of this data complies with the terms and conditions set by the respective UN agencies, eliminating the need for formal permission to access and analyse the datasets. More information about the data sources can be found in the “Materials and Methods” section.
